# Small Bowel Obstruction Secondary to Partial Malrotation of the Gut: A Case Report

**DOI:** 10.7759/cureus.77031

**Published:** 2025-01-06

**Authors:** Alec K Donohue, Ilya V Latyshenko, Lawrence F Sugden, Ryan M Kozloski, Jason C McCartt

**Affiliations:** 1 General Surgery, Womack Army Medical Center, Fort Liberty, USA; 2 Internal Medicine, Womack Army Medical Center, Fort Liberty, USA

**Keywords:** general emergency surgery, internal abdominal hernia, left paraduodenal hernia, midgut malrotation, partial malrotation

## Abstract

This case report presents a unique clinical presentation of small bowel obstruction secondary to congenital partial malrotation of the gut in adults. Partial malrotation may have variable clinical presentations and this case highlights a constellation of patient history, radiographic signs, and operative findings leading to appropriate diagnosis and successful surgical management. A 56-year-old female patient presented with severe abdominal pain, nausea, and anorexia. She reported acute on chronic vague, intermittent cramping abdominal pain for approximately five months. Prior evaluations of her gastrointestinal symptoms did not reveal a clear etiology. On examination, the patient’s vital signs were within normal limits and the abdominal exam was benign. Computed tomography (CT) of the abdomen and pelvis with IV contrast, obtained in the emergency department, was concerning for paraduodenal hernia but was also notable for the lack of a duodenal sweep. With the severity of abdominal pain being out of proportion to the physical exam and the aforementioned radiographic findings, we decided to proceed with diagnostic laparoscopy. Intraoperative findings included a Ladd band forming a potential space for the incarceration of the bowel, internal herniation of the partially reducible small bowel, and a narrow mesenteric base. A laparoscopic Ladd procedure was performed and the patient recovered without complications. Her chronic gastrointestinal complaints have abated since the operative intervention. This case underscores the importance of considering a rare diagnosis such as partial malrotation of the gut in adults presenting with acute on chronic abdominal pain, the key clinical features associated with this pathology, and its successful operative management. Furthermore, this case highlights the importance of early recognition and management to minimize the morbidity and mortality of devastating sequelae such as midgut volvulus and closed-loop obstruction.

## Introduction

Congenital malrotation of the gut results from improper rotation of the physiologically herniated midgut between the gestational weeks of seven and 12 [1]. Although typically diagnosed in infancy, the condition may present in adults with a wide array of symptoms [2,3]. This case report describes the unique presentation of small bowel obstruction secondary to partial malrotation of the gut in a 56-year-old female patient. Successful surgical management involved the reduction of the herniated bowel, addressing the anatomical abnormalities while allowing for internal herniation, and completion of the Ladd procedure.

## Case presentation

**Figure 1:**
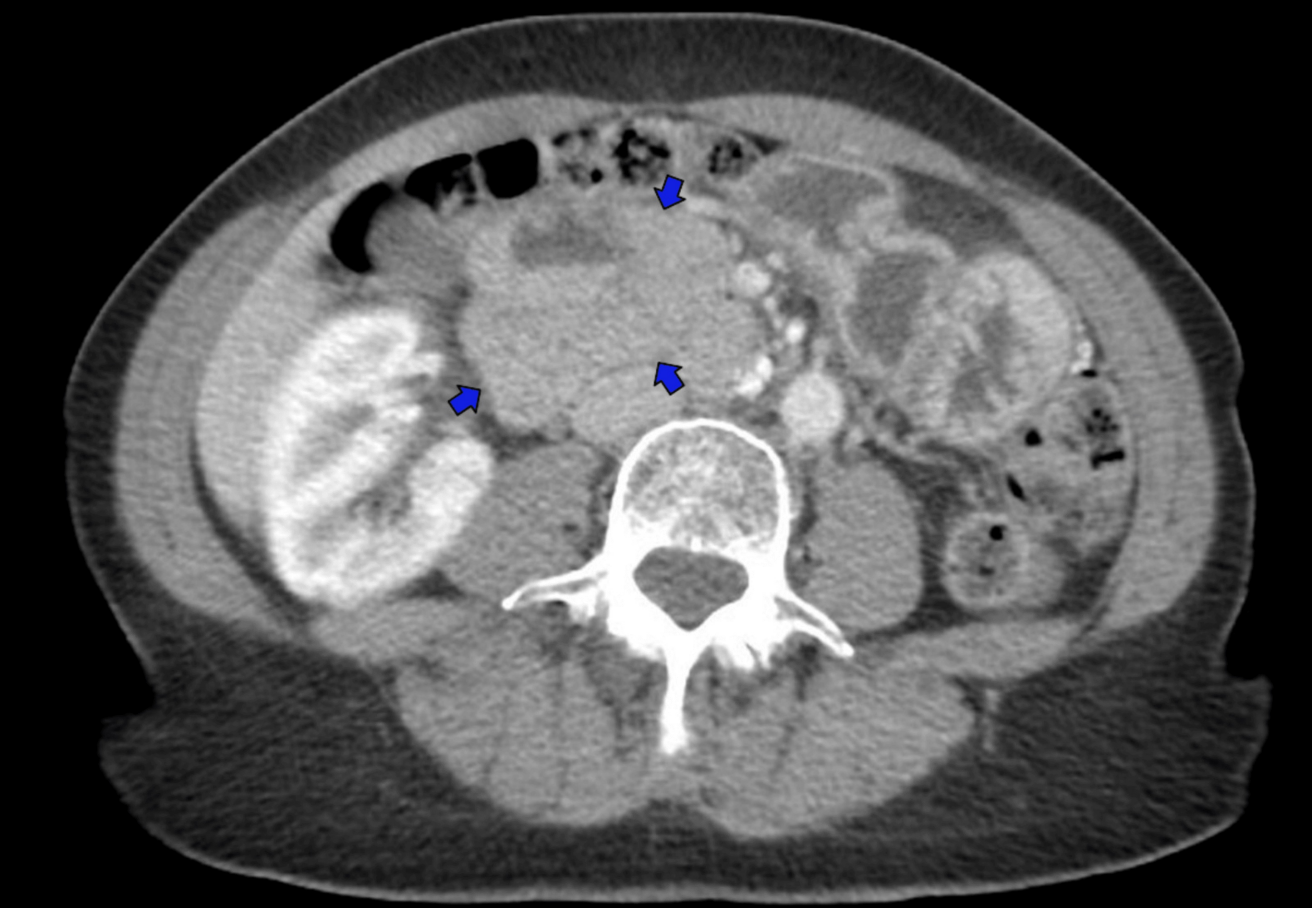
Axial CT of the abdomen and pelvis demonstrating a right upper quadrant hernia containing the small bowel (blue arrows)

**Figure 2:**
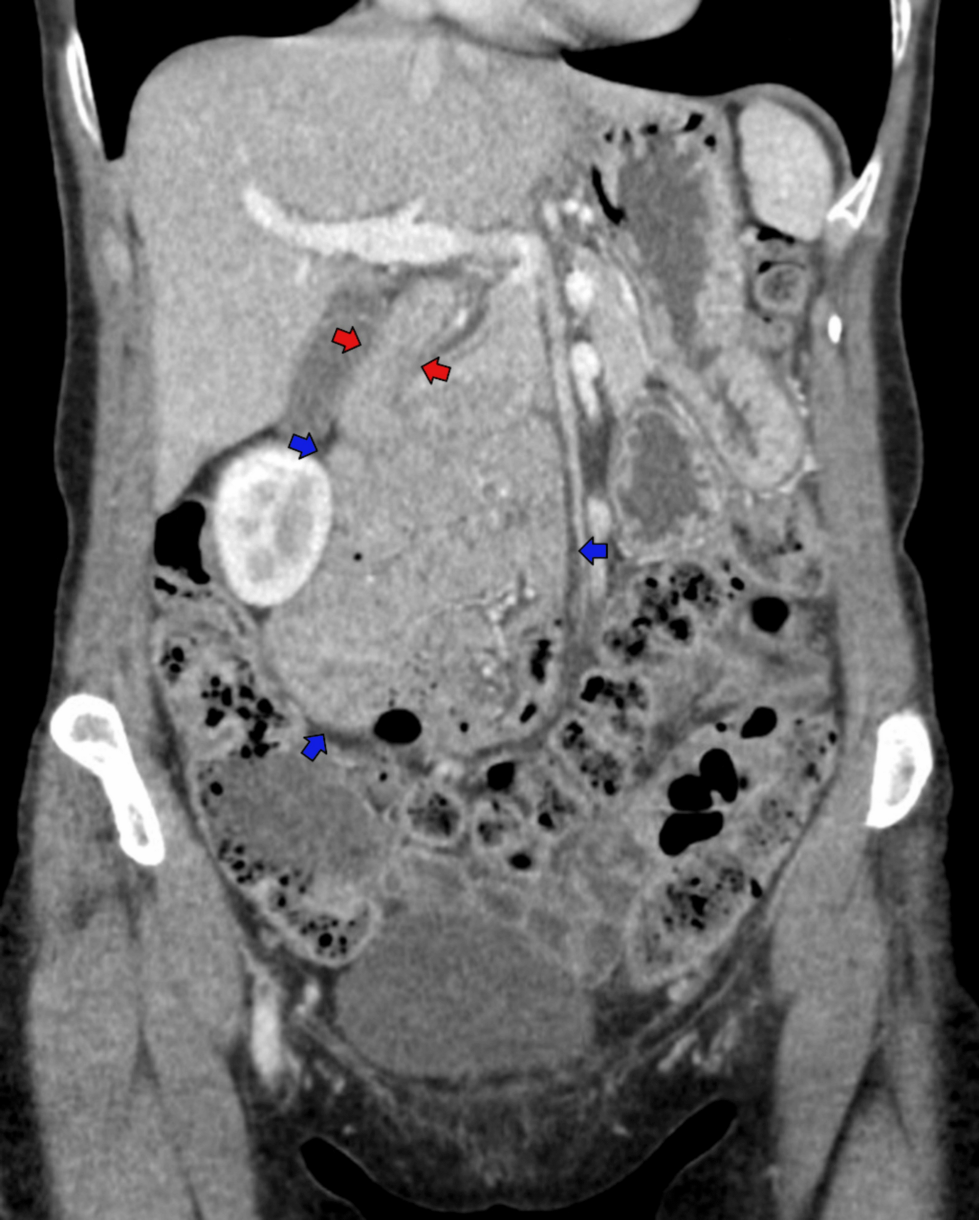
Coronal CT of the abdomen and pelvis demonstrating inferior trajectory of the duodenum and a lack of duodenal sweep (red arrows) and herniation of the majority of the small bowel into right upper quadrant (blue arrows)

A 56-year-old female patient presented to the emergency department with severe, unrelenting abdominal pain characterized as cramping with intermittent sharpness in the epigastric region. She additionally reported nausea without vomiting and anorexia. The patient noted that her last bowel movement was the evening before the presentation, but could not recall the last time she had passed flatus. She reported experiencing vague, intermittent cramping abdominal pain for approximately five months and having had one previous episode severe enough to warrant evaluation in the emergency department. During her earlier evaluation, abdominal radiographs were performed which did not demonstrate any obvious pathology explaining her symptoms. She was subsequently discharged with instructions regarding dietary modification and was asked to follow up with her primary care physician. Twelve days following her discharge from the previous emergency department evaluation, the patient presented again with recurrent vague abdominal pain. These symptoms were unique in that she noted an increasing frequency of severe pain episodes accompanied by chills, nausea without vomiting, and anorexia. The patient remarked that her last bowel movement was the evening before her presentation. Her medical and surgical history was significant only for gastroesophageal reflux and a C-section done in 1986. On physical examination, the patient was hemodynamically normal and her abdomen was soft, nondistended, and mildly tender with deep palpation. Laboratory studies performed in the emergency department showed no abnormalities. However, CT of the abdomen and pelvis was concerning for internal hernia and abnormal orientation of the small intestine with pylorus, duodenum, and jejunum located in the right upper quadrant (Figures 1, 2).

Given the patient's symptoms, pain out of proportion to her exam, and abnormal imaging findings, diagnostic laparoscopy was performed due to concern for internal hernia with a possibly ischemic gut.

**Figure 3:**
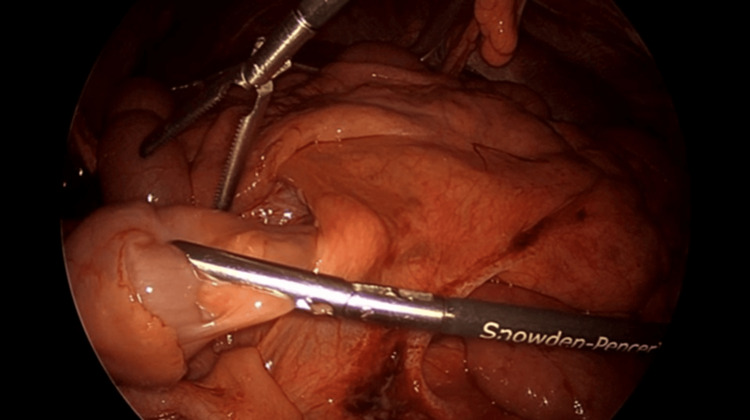
Internal herniation of the small bowel through the space created by the Ladd band

Entry into the abdomen was accomplished using a direct optical trocar. Three 5 mm ports were placed in the infraumbilical, supra-pubic and lateral left lower quadrant. The cecum was identified, and small bowel was evaluated from distal to proximal until the site of the internal hernia was quickly identified (Figure 3).

**Figure 4:**
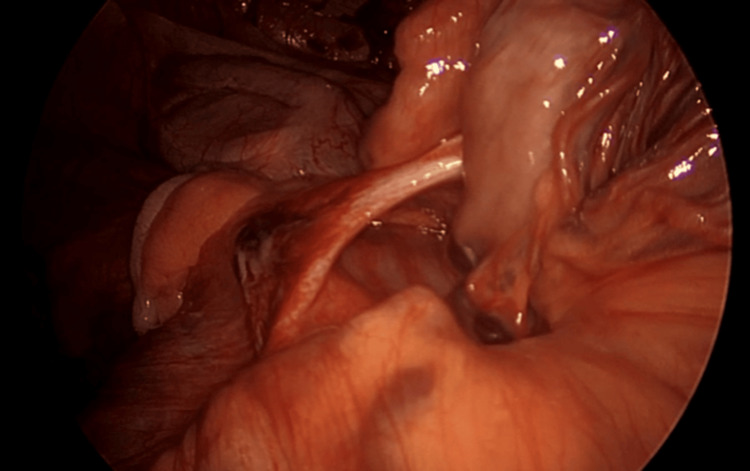
A single Ladd band forming a potential space for herniation

A dense fibrotic band, consistent with a Ladd band, allowed for the creation of a potential space for herniation (Figure 4).

**Figure 5:**
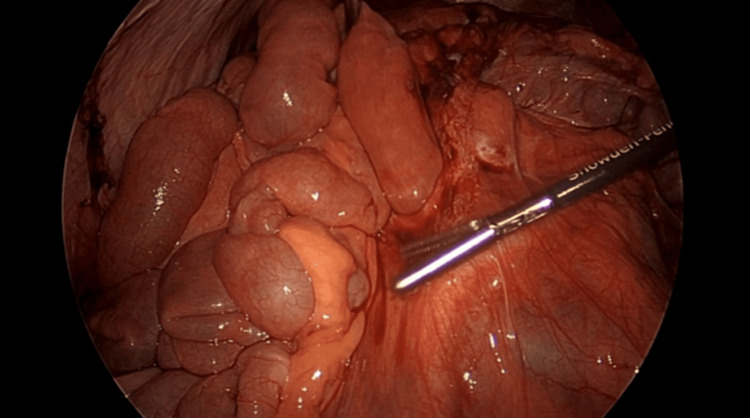
Reduced small bowel appearing viable and well perfused

The majority of the herniated small bowel was easily reduced and appeared grossly viable and well perfused (Figure 5).

**Figure 6:**
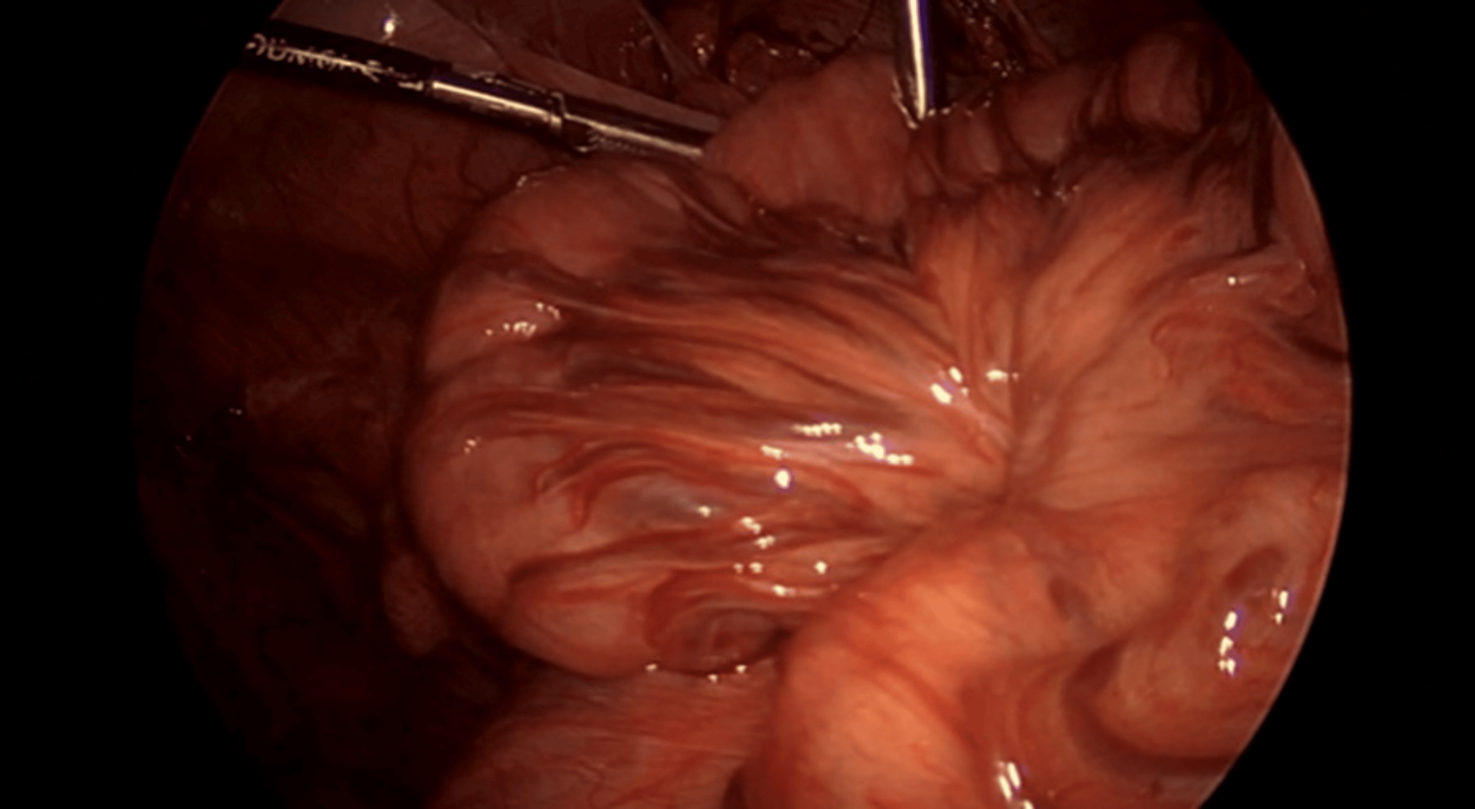
Narrow mesenteric base as classically described in congenital malrotation and variants

The Ladd band was divided using a laparoscopic bipolar energy device (Covidien LigaSure ™ Blunt Tip without Nano-Coating Sealer/Divider, Medtronic, Minneapolis, MN) and the remaining incarcerated bowel was freed and inspected. Beginning at the cecum, the bowel was again evaluated from ileocecal valve to the ligament of Treitz revealing a narrow mesenteric base (Figure 6).

**Figure 7:**
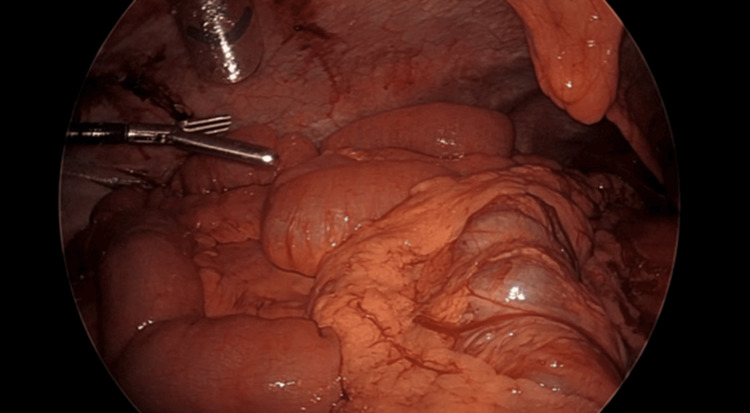
Post-operative anatomic arrangement with the small bowel residing in the patient’s right hemi-abdomen and colon in the left

The entire small bowel appeared viable and no additional Ladd bands or sites for re-herniation were identified. Given that the patient's preoperative imaging and the intraoperative findings were consistent with partial malrotation, we made the decision to complete the Ladd procedure. A laparoscopic right medial visceral rotation and appendectomy were completed allowing the small bowel to reside in the right hemi-abdomen and the colon in the left hemi-abdomen (Figure 7).

The patient progressed appropriately after the procedure and reported resolution of the pain associated with her acute on chronic symptoms. The patient was seen in the clinic two weeks following discharge and at three months for continuity and there has been no recurrence of her previous gastrointestinal complaints.

## Discussion

During normal fetal development, the midgut develops rapidly leading to physiologic herniation between gestational weeks seven and 12. Before spontaneous reduction into the abdominal cavity, the midgut undergoes a 270-degree counterclockwise rotation [1]. Congenital malrotation occurs when this normal rotational process fails [2]. This failure is marked by a narrow mesenteric base and the presence of Ladd bands. Imaging will often reveal associated features including a lack of duodenal sweep and a superior mesenteric vein oriented left of the superior mesenteric artery [2,3]. Herniation, rotation, and reduction occur over several weeks during embryogenesis, allowing for multiple pathological variants including incomplete (partial) rotation, non-rotation, and reverse rotation [3].

Malrotation is often diagnosed and treated within the first year of life, and as such, its exact prevalence in the adult population is unknown [4]. Adult patients with malrotation often present with chronic symptoms such as intermittent abdominal pain, bloating, vomiting, and food intolerance. Many of them have undergone extensive workups without the identification of a clear etiology or explanation of their symptoms, and have often failed multiple treatment attempts [2,3]. Acute symptoms, although less common, can include profuse nausea and vomiting, hematemesis, and peritonitis. Patients presenting with acute symptoms may indicate the occurrence of the severe sequelae of the frequently missed diagnosis of malrotation. These emergent sequelae include internal hernia with associated bowel obstruction, midgut volvulus, and perforation [5,6].

In this case, a 56-year-old female patient presented with acute on chronic diffuse abdominal pain, nausea, and anorexia. Despite previous workup and one prior emergency department evaluation, a clear source for her ongoing abdominal complaints had not been identified. CT performed in the emergency department revealed a lack of duodenal sweep and a normal anatomic orientation of the superior mesenteric artery and vein with a concern for internal hernia. Given these findings and with severe pain out of proportion to the patient’s abdominal exam, we decided to perform diagnostic laparoscopy. Intraoperatively, an internal hernia was identified through the pathological space created by a Ladd band. After ensuring the viability of the bowel, the Ladd procedure was completed. The patient recovered appropriately with complete resolution of her previous gastrointestinal complaints.

## Conclusions

This case underscores the importance of considering congenital malrotation in patients presenting with chronic abdominal pain. Midgut volvulus is one of the classically described and feared sequelae of this congenital abnormality. However, a variation in the degree of malrotation provides an opportunity for other devastating consequences including an internal hernia and a closed-loop bowel obstruction. Understanding the types of malrotation variants and their possible clinical sequelae ensures an appropriate index of suspicion in patients with difficult-to-diagnose chronic abdominal pain. Early identification and treatment may serve to minimize the morbidity and mortality associated with the devastating sequelae related to the congenital malrotation of the gut.
